# The use of spirituality/religiosity by oncology nurse residents in nursing care

**DOI:** 10.1590/0034-7167-2023-0383

**Published:** 2024-07-19

**Authors:** Ana Paula Kelly de Almeida Tomaz, Rômulo Frutuoso Antunes, Rachel Verdan Dib, Raquel de Souza Ramos, Flaviana Pereira Bastos Nascimento, Sérgio Abreu de Jesus, Kayo Henrique Jardel Feitosa Sousa, Regina Célia Gollner Zeitoune

**Affiliations:** I Instituto Nacional de Câncer. Rio de Janeiro, Rio de Janeiro, Brazil; II Hospital Israelita Albert Einstein. São Paulo, São Paulo, Brazil; III Universidade do Estado do Rio de Janeiro. Rio de Janeiro, Rio de Janeiro, Brazil; IV Universidade Federal do Rio de Janeiro. Rio de Janeiro, Rio de Janeiro, Brazil

**Keywords:** Spirituality, Religion, Complementary Therapies, Oncology Nursing, Internship, Nonmedical, Espiritualidad, Religión, Terapias Complementarias, Enfermería Oncológica, Internado no Médico

## Abstract

**Objectives:**

to analyze the use of spirituality/religiosity by oncology nurse residents in caring for patients with cancer.

**Methods:**

a census, descriptive, sectional study, with 46 nurse residents from three public hospitals in Rio de Janeiro. Data collection took place between August 2020 and January 2021, using a sociodemographic questionnaire, including a question about the use of spirituality/ religiosity to deal with work situations. Descriptive analysis was carried out using SPSS software version 22.0.

**Results:**

participants stated that they use religiosity/spirituality in work situations related to patients or themselves. In relation to patients, death was the most mentioned situation among professionals, and for themselves, everyday situations and emotional vulnerability were the most mentioned.

**Final Considerations:**

spirituality and religiosity are dimensions that guide oncology nurse residents’ attitudes.

## RESUMEN

**Objetivos:**

analizar el uso de la espiritualidad/religiosidad por parte de enfermeras residentes de oncología en el cuidado del paciente con cáncer.

**Métodos:**

estudio censal, descriptivo, seccional, con 46 enfermeros residentes de tres hospitales públicos de Río de Janeiro. La recolección de datos se realizó entre agosto de 2020 y enero de 2021, mediante un cuestionario sociodemográfico, que incluía una pregunta sobre el uso de la espiritualidad/religiosidad para afrontar situaciones laborales. El análisis descriptivo se realizó mediante el *software* SPSS, versión 22.0.

**Resultados:**

los participantes afirmaron utilizar la religiosidad/espiritualidad en situaciones laborales relacionadas con el cliente o con ellos mismos. En relación al cliente, la muerte fue la situación más mencionada entre los profesionales, y para usted, las situaciones cotidianas y su vulnerabilidad emocional fueron las más mencionadas.

**Consideraciones Finales:**

espiritualidad y religiosidad son dimensiones que orientan las actitudes de los enfermeros residentes en oncología.

## INTRODUCTION

Nursing residency programs are part of *lato sensu* graduate education, also called specialization. They are characterized by in-service teaching, with a weekly workload of 60 hours dedicated to theoretical, practical and theoretical-practical activities, on an exclusive dedication basis, lasting two years. This period of weekly work provides a basis for professionals’ care practice, and at the end, with compliance with the minimum learning and working time requirements, residents obtain a specialist title in the chosen area^(1)^.

During the residency period, students deal directly with the patient in their practical and theoretical-practical activities. Each user presents their singularities, such as different beliefs, knowledge, cultures and values that form the way of dealing with the disease and, likewise, the professionals who use them to provide nursing care. From this perspective, spirituality/religiosity have dimensions that constitute the human population’s cultural, social and moral experience, being understood as the relationship with the sacred and the transcendent, originating or not religious communities and rituals^(2)^.

Spirituality is understood as the multiple human dimensions that are expressed through behaviors, feelings and relationships, being linked to the set of convictions and experiences that reflect the care one has for life^(3)^. It is integrated with the belief in a Higher Being that brings humans closer to what elevates their mortal condition to the situation of immortality, in the search for comfort and the perspective of change^(3)^. Spirituality occurs through finding meaning in life, attributing purpose to it based on the connection with a Higher or Transcendent Being, and is not necessarily associated with religious practice^(4-5)^.

With regard to religiosity, it is linked to the belief and practice of a religion, whether through going to church, religious temple, prayers, reading, symbols or images and rites, with the aim of bringing individuals closer to a Higher Being^(5-6)^. Religiosity can be used as a support tool for daily coping with human existence subjectivized by living with a chronic and stigmatized condition such as cancer^(7)^.

In this study, we chose to use the terms “spirituality” and “religiosity” combined (spirituality/religiosity), considering that it was not the objective of this to identify issues intrinsic to each of these dimensions, but the way in which residents deal with what transcends the more objective issues of nursing care in oncology hospital units.

In oncology, with regard to illness, having cancer is having a disease that, despite great technological advances and the achievement of a higher cure rate, is still linked in common sense to finitude and negative feelings, such as sadness, due to its high incidence as well as side effects related to treatment^(8)^. Such feelings are not only experienced by patients who present the disease, but also by the multidisciplinary team that provides care.

Thus, offering conditions for the spiritual/religious manifestation of patients within the hospital environment has a significant impact on their overall quality of life, as they enjoy and have their values and beliefs respected. Thus, the importance of religiosity/spirituality in the evolution of individuals’ clinical condition and their support network is reinforced, reflecting on the way they view the process of illness and finitude^(9)^. This manifestation can also occur among nursing professionals, in the search for tools to offer comfort and ways to deal with patients’ distress in the possibility of preserving their physical and mental health, consequently mitigating work-related damages resulting from psychosocial factors^(10)^.

A study with undergraduate nursing students demonstrated that religiosity is considered an important ally in the process of accepting and coping with death, and can be a great influence on individuals’ conceptions of the death and dying process, influencing nurses’ attitudes in a Christian context, as they see death as a natural part of life or as a portal to life after death, directly influencing the care provided^(10)^.

The World Health Organization (WHO) defined health as beyond the absence of disease. It is a combination of physical, psychological, social and spiritual well-being. It is evident, therefore, that spirituality must be recognized as part of care and addressed in healthcare professional training^(11-12)^. Considering the relevance of the topic and its importance for resident training, the literature review carried out showed that the spiritual dimension, when considered within the scope of oncological care, strengthens the emotional bonds between patient-professional-companion, in addition to generating meaning in work for healthcare professionals^(13)^.

Therefore, to transform professional practice, it is urgent that the topic of spirituality/religiosity be included in the curricula of undergraduate and graduate courses so that students recognize the indispensability of the topic in their field of activity, expanding the understanding of spirituality and religiosity, and their role for both patients and healthcare professionals, seeking comprehensive and humanized care^(11)^ and support for workers’ health. Therefore, the study is justified because spirituality and religiosity are considered as a strategy for coping with life.

## OBJECTIVES

To analyze the use of spirituality/religiosity by oncology nurse residents in caring for oncology patients.

## METHODS

### Ethical aspects

This study respected the ethical precepts regarding research involving human beings, as recommended by Resolution 466/12 of the Brazilian National Health Council. The study was approved by the Research Ethics Committee of the proposing institution and by the co-participating institution. The Informed Consent Form (ICF) was made available to participants in writing in two copies.

### Design and setting

This is a census, descriptive, sectional study. The study site was three public hospitals located in the state of Rio de Janeiro. All institutions had treatments aimed at people living with cancer as well as a uni or multidisciplinary oncology residency program.

### Population or sample; inclusion and exclusion criteria

A total of 46 residents participated in the research, equivalent to 100% of the population, regardless of year of course (first or second), enrolled in an oncology residency program. Inclusion criteria included nurse residents enrolled in an oncology residency program in the state of Rio de Janeiro in the first or second year of the program. Exclusion criteria included residents with less than six months in the aforementioned program due to the period of theoretical classes, without direct contact with patients, or who were away on leave for health treatment.

### Outcome measures

To identify the sociodemographic profile of nurse residents, a self-administered questionnaire was used, prepared by the authors, based on studies that used the Psychosocial Risk Assessment Protocol at Work. The issues that constituted the study variables were sex, age, marital status, place of origin, religious belief and whether participant used spirituality/religiosity in care practice and, if so, in what work situation.

### Data collection

Data collection took place between August 2020 and January 2021, respecting all safety standards regarding COVID-19 guided by the WHO and Pan American Health Organization (PAHO)^(14)^. Data collection took place in person, using a sociodemographic questionnaire, including a question about the use of spirituality and religiosity to deal with work issues. The questionnaires were completed by study participants after reading, accepting and signing the ICF.

### Statistical analysis

To analyze the collected data, Statistical Package for the Social Sciences (SPSS) version 22.0 was used. Relative and absolute frequencies were used to analyze the sociodemographic profile of participants and to analyze data regarding spiritual and religious practices. The reasons for religious/spiritual practices were distributed according to religious belief.

## RESULTS

Regarding the profile of residents (Table 1), the majority were female (91.3%), with a predominance of ages between 23 and 29 years (78.3%), and the majority declared to be single (80.4%). As for origin, 73.9% of participants came from the Southeast. Regarding religion, Protestantism (34.8%) and Catholicism (32.6%) stood out, and when asked about the use of spirituality/religiosity in work situations, the majority said they use it in their work environment (76.1%).

**Table 1:** Sociodemographic characteristics of nurse residents in oncology residency programs in the state of Rio de Janeiro, Rio de Janeiro, Rio de Janeiro, Brazil, 2020 (N=46)

Variables	n	%
Age (years)		
23-29 years	36	78.26%
30-36 years	8	17.39%
37-44 years	2	4.35%
Sex		
Female	42	91.30%
Male	4	8.70%
Origin (country region)		
Southeast	34	73.92%
Northeast	10	21.73%
Center-West	2	4.35%
Marital status		
Married	6	13.04%
Divorced	1	2.17%
Single	37	80.43%
Stable union	2	4.35%
Religion		
Catholicism	15	32.6%
Protestantism	16	34.8%
Spiritism	4	8.7%
African religions	6	13.0%
Without religion	5	10.9%
Do you use spirituality/religiosity to deal with your work issues?		
Yes	35	76.10%
No	11	23.90%

In relation to situations in which residents used spirituality/religiosity in work situations, there was reference to situations related to patients (death, fear, mourning and distress, finitude and coping with the disease) and those related to themselves (in all daily situations, emotional vulnerability, fear of death and illness and different feelings), as shown in Table 2.

**Table 2:** Distribution of responses regarding situations in which residents used religiosity and spirituality to provide care to patients with cancer (n=35)^*^, Rio de Janeiro, Rio de Janeiro, Brazil, 2022

Moments of nurse residents’ care practice	Religion				
	Catholic	Protestant	Spiritism/Afro	No religion	Other
Situations related to patients					
Patient death	4	2	1	4	3
Fear reported by patients	1	1		1	
Mourning and distress				1	
Finitude		1			1
Coping with the disease	1				
Situations related to themselves					
All situations (daily difficulties/difficult situations)	2	5	1		
Emotional vulnerability, concern and stress	1	3		1	1
Request for help	1	1			
Unexpected event (patient death)	1				1
Fear of own death (self-illness)	1	1			
Strength to continue		1			
Feeling of joy		1			
Feeling of sadness		1			
Feeling of anger		1			
Feeling of hurt	1				

* *Participants could have multiple responses.*

## DISCUSSION

Spirituality and religiosity are interfaces that go hand in hand, and people who use them have better general well-being, with lower rates of anxiety, depression and risk behaviors, such as suicide and abusive use of psychoactive substances^(15-16)^. In the same study^(15)^, spirituality encompasses the connection, which may or may not be religious, with a non-physical reality, as perceived by individuals^(16)^.

A study carried out in the Netherlands indicated spirituality and religion as essential elements for healthcare, in agreement with the present study, pointing out that both religion and spirituality can influence patients’ and healthcare professionals’ lives. At the international study location, the spiritual and religious dimensions are vital for health action^(17)^.

It is observed that the spiritual and religious dimension brings constructive emotions and, when experienced in a positive way, helps patients to face the disease. Hence, it contributes to physical and mental state preservation and, in addition to self-care, reducing the stress and anxiety generated by treatment in patients with cancer^(18)^.

In everyday life, with residents, it is possible to observe that, in addition to the complexity of caring for oncology patients, they still have the challenge of dealing with their own emotional, spiritual and religious burdens, also experiencing reflections about their training and professional performance in care practice^(19)^.

A study^(20)^ highlighted the importance of the applicability of spirituality and religiosity in clinical practice, revealing that they provide strength and confidence to patients with neoplasia to face difficult situations throughout the process of seeking a cure, pointing out the essentiality of using faith in this phase. These results reinforce that it is increasingly necessary to know the spiritual care demands of these people. Such data support the study in question that identified a predominance of the use of spirituality/religiosity in healthcare activities by nurse residents. Furthermore, most individuals use spirituality to deal with emotional vulnerability, psychological distress and mental changes associated with anxiety and other negative feelings^(16)^.

Spirituality/religiosity are a dimension that transcends carnal planes and guides the attitudes of individuals. A cross-sectional study, carried out at hospitals in Rio de Janeiro that had oncology residency programs^(3)^ with 53 professionals working with critically ill patients, revealed that the majority had high spirituality and that this dimension had a strong influence on the health-disease process understanding and management. Furthermore, it was found that spirituality/religiosity were capable of promoting a state of calm and tranquility as well as improvements in the social, emotional and behavioral dimensions of those who exercised it.

American research has indicated that positive spiritual experiences can improve a person’s perception of their self-rated health, and can be used as functional and positive tools by healthcare professionals to promote well-being^(21)^. Other evidence shows that spirituality is strongly related to dealing with stressful moments, while religiosity is aimed at better health indicators such as better quality of life, reduced rates of depression, suicide and drug use^(22)^. Spirituality and religiosity are essential factors in healthcare, as they contribute positively from the diagnosis of a disease that goes through delicate moments and its coping both by individuals and their support network, contributing to adherence to the proposed therapy^(23)^.

A cross-sectional, descriptive and quantitative study, with 31 nurses who worked for at least six months in a teaching hospital in the state of São Paulo, carried out using a convenience sample, showed that participants reported that spirituality and religiosity interfere in several aspects of human beings, such as health. Additionally, 87% of participants reported feeling the presence of God or the Holy Spirit. Combined with this data, 97% of nurses declared that they believe in God. In this same study, 45% of nurses who participated in the research described spirituality as the search for meaning in their existence, highlighting its essentiality^(24)^.

Descriptive, quantitative-qualitative research revealed that 57.6% (n=85) of healthcare professionals considered themselves very or completely spiritual. Furthermore, they believed that religion could have positive effects on sick individuals’ health. Furthermore, employees’ perception of the influence of spirituality and religiosity focuses on the idea of being a “source of comfort” and strengthening in dealing with patients with poor prognoses^(13)^, i.e., with a small probability of improvement.

English and Italian researchers pointed out that nurses regularly provided spiritual care to patients, and that this role depends on professionals’ awareness and involvement with the topic. Those who received training were able to provide care that increased patients’ trust on professionals. With regard to patients with cancer, a positive impact was considered as a result^(25)^.

The research revealed that care strategies associated with spirituality in the face of the death process of patients with cancer had multidimensional and relational meaning, as they encompass meanings, life goals, meanings, beliefs and self-reflection, which can be observed through active listening, words of comfort and affection in the face of the finiteness of life^(13)^.

An exploratory cross-sectional study with 55 residents in a transversal module on spirituality and integrality showed that 65.45% of participants considered it necessary to prepare the resident in spirituality and that 38.18% read sacred books, prayers and prayers daily^(26)^. Through teaching, it is possible to adopt scientific knowledge about spirituality and religiosity in the health context, learning and practicing in the care provided to patients, as was found in a Brazilian study, which analyzed the use of spirituality in the resident training process, identifying the relevance of conceptual use associated with practice to add positive values to the nursing care provided to patients^(26)^.

### Study limitations

The limitations of this study are associated with the fact that it was carried out only from the perspective of graduate students and in a single specialty, oncology. It is suggested that further research be developed to understand students’ perception of the relevance of including the topic in undergraduate and graduate curricula as possibilities to provide professionals with the necessary tools and qualify care from this perspective with patients and in view of their needs.

The number of participants and the type of census study can be considered a limitation; therefore, more studies are needed to support or refute the findings presented. It is also suggested to carry out multicentric research in order to broaden the reflection on the use of spirituality and religiosity in residents’ practice.

This research had as a limitation the small number of publications on the topic among nurse residents, which made it difficult to compare results with other realities. This reveals the pressing need for investments in scientific production in this field, in order to qualify spirituality and religiosity as tools for nursing care.

### Contributions to nursing, health, or public policies

The need to include this topic in curricular matrices of higher education courses in different professions in health is highlighted so that these professionals can adopt, with more accuracy and scientific basis, spirituality and religiosity in their therapeutic care plans. Furthermore, the adoption of spirituality and religiosity in the workplace can contribute to better coping in difficult situations.

## FINAL CONSIDERATIONS

The use of spirituality and religiosity in a hospital environment brings a series of benefits to patient-professional-companion, from better acceptance and coping with the disease to the reality of finitude as well as the preservation of the mental and physical health of healthcare professionals who experience this scenario frequently. Spirituality/religiosity are mechanisms used by nurse residents to deal with situations experienced by patients, complex work demands as well as for self-reflection on care practices.

The situation with the greatest applicability of spirituality/religiosity reported by residents is patient death, regardless of religious belief, since finitude and mourning can be seen as failures of healthcare, as academic training in health still mirrors the role of the biomedical model. Furthermore, the benefit is mainly aimed at difficult and challenging situations for residents, when they cling to religiosity and spirituality to alleviate their psychological and emotional distress.

Therefore, the implementation of the topic of spirituality/religiosity since graduation as well as its continuity in graduate programs becomes essential given the positive results that its practice presents.
