# Supplementary material for: The use of spirituality/religiosity by oncology nurse residents in nursing care
Source: Rev Bras Enferm. 2024 Jul 19;77(2):e20230383. doi: 10.1590/0034-7167-2023-0383 (PMC11259435; doi:10.1590/0034-7167-2023-0383)
Supplement: Translation

# Translation: O uso da espiritualidade/religiosidade por enfermeiros residentes em oncologia na assistência de enfermagem

**DOI:** 10.1590/0034-7167-2023-0383pt

**Authors:** Ana Paula Kelly de Almeida Tomaz, Rômulo Frutuoso Antunes, Rachel Verdan Dib, Raquel de Souza Ramos, Flaviana Pereira Bastos Nascimento, Sérgio Abreu de Jesus, Kayo Henrique Jardel Feitosa Sousa, Regina Célia Gollner Zeitoune

**Affiliations:** I Instituto Nacional de Câncer. Rio de Janeiro, Rio de Janeiro, Brasil; II Hospital Israelita Albert Einstein. São Paulo, São Paulo, Brasil; III Universidade do Estado do Rio de Janeiro. Rio de Janeiro, Rio de Janeiro, Brasil; IV Universidade Federal do Rio de Janeiro. Rio de Janeiro, Rio de Janeiro, Brasil

**Keywords:** Espiritualidade, Religião, Terapias Complementares, Enfermagem Oncológica, Internato não Médico

## Abstract

**Objetivos:**

analisar o uso da espiritualidade/religiosidade por enfermeiros residentes em oncologia na assistência ao paciente oncológico.

**Métodos:**

estudo censitário, descritivo, do tipo seccional, com 46 enfermeiros residentes de três hospitais públicos do Rio de Janeiro. A coleta de dados ocorreu entre agosto de 2020 e janeiro de 2021, utilizando um questionário sociodemográfico, incluindo uma pergunta acerca do uso da espiritualidade/religiosidade para lidar com situações do trabalho. A análise descritiva foi realizada por meio do *software* SPSS, versão 22.0.

**Resultados:**

os participantes afirmaram fazer uso da religiosidade/espiritualidade em situações de trabalho relacionadas ao cliente ou a si próprio. Em relação ao cliente, a morte foi a situação mais referida entre os profissionais, e para si, as situações do dia a dia e sua vulnerabilidade emocional foram as mais referidas.

**Considerações Finais:**

a espiritualidade e a religiosidade são dimensões que guiam as atitudes dos enfermeiros residentes em oncologia.

## INTRODUÇÃO

Os programas de residência em enfermagem constituem-se ensino de pós-graduação *lato sensu*, também denominada especialização. São caracterizados por ensino em serviço, com carga horária semanal de 60 horas destinada às atividades teóricas, práticas e teórico-práticas, em regime de dedicação exclusiva, com duração de dois anos. Esse período de trabalho semanal permite embasar a prática assistencial dos profissionais, e ao final, com o cumprimento dos requisitos mínimos de aprendizado e de tempo de trabalho, o residente obtém um título de especialista na área escolhida^(1)^.

Durante o período da residência, o discente lida diretamente com o paciente em suas atividades práticas e também teórico-práticas. Cada usuário apresenta suas singularidades, tais como diferentes crenças, saberes, culturas e valores que formam a maneira de lidar com a doença e, da mesma forma, os profissionais que as utilizam para prestar cuidados de enfermagem. Nessa perspectiva, tem-se na espiritualidade e na religiosidade dimensões que constituem a experiência cultural, social e moral da população humana, sendo entendidas como a relação com o sagrado e o transcendente, originando ou não comunidades e rituais religiosos^(2)^.

Compreende-se por espiritualidade as múltiplas dimensões humanas que se externam através de comportamentos, sentimentos e relacionamentos, estando ligada ao conjunto de convicções e às experiências que refletem o cuidado que se tem com a vida^(3)^. Integra-se à crença em um Ser superior que aproxima o humano daquilo que eleva a sua condição mortal à situação de imortalidade, na busca de conforto e perspectiva de mudança^(3)^. A espiritualidade se dá por meio do encontro de sentido pela vida, atribuindo propósito à mesma a partir da conexão com um Ser superior ou transcendente, não estando necessariamente associada à prática religiosa^(4-5)^.

No que se refere à religiosidade, essa se encontra interligada à crença e à prática de uma religião, seja por meio de idas à igreja, templo religioso, orações, leitura, símbolos ou imagens e ritos, com a finalidade de aproximação do indivíduo com um Ser superior^(5-6)^. A religiosidade pode ser usada como uma ferramenta de apoio de enfrentamento cotidiano relativo à existência humana subjetivado pela convivência com uma condição crônica e estigmatizada como o câncer^(7)^.

Neste estudo, optou-se por utilizar os termos “espiritualidade” e “religiosidade” de forma combinada (espiritualidade/religiosidade), considerando que não foi objetivo deste identificar questões intrínsecas a cada uma dessas dimensões, mas a forma como os residentes lidam com o que transcende as questões mais objetivas do cuidar em enfermagem em unidades hospitalares oncológicas.

No contexto da oncologia, no que se refere ao adoecimento, ter câncer é ter uma doença que, apesar do grande avanço tecnológico e do alcance de maior índice de cura, ainda se encontra atrelada, no senso comum, à finitude e a sentimentos negativos, como tristeza, devido à sua alta incidência, bem como aos efeitos colaterais relacionados ao tratamento^(8)^. Tais sentimentos não são experienciados somente pelo cliente que apresenta a doença, mas também pela equipe multidisciplinar que presta os cuidados.

Assim, oferecer condições para a manifestação espiritual/religiosa dos pacientes dentro do ambiente hospitalar repercute de forma significativa na qualidade de vida global desses, pois desfrutam e têm seus valores e crenças respeitados. Dessa maneira, reforça-se a importância da religiosidade e espiritualidade na evolução do quadro clínico do indivíduo e de sua rede de apoio, refletindo no modo como encaram o processo de adoecimento e finitude^(9)^. Essa manifestação pode acontecer também entre os profissionais de enfermagem, perante a busca de ferramentas para oferecer conforto e formas de lidar com o sofrimento do paciente na possibilidade de preservar sua saúde física e mental, consequentemente mitigando os danos relacionados ao trabalho decorrentes de fatores psicossociais^(10)^.

Estudo com estudantes de graduação em enfermagem demonstrou que a religiosidade é considerada uma importante aliada no processo de aceitação e enfrentamento da morte, e pode ser uma grande influenciadora nas concepções sobre o processo de morte e morrer dos indivíduos, influenciando atitudes dos enfermeiros em um contexto cristão, pois veem a morte como parte natural da vida ou como um portal para a vida após a morte, influenciando diretamente o cuidado prestado^(10)^.

A Organização Mundial da Saúde (OMS) definiu saúde como além da ausência de enfermidades. Trata-se de uma combinação de bem-estar físico, psíquico, social e espiritual. Evidencia-se, dessa forma, que a espiritualidade deve ser reconhecida como parte do cuidado e abordada na formação dos profissionais de saúde^(11-12)^. Considerando a relevância da temática e sua importância para a formação do residente, a revisão de literatura realizada evidenciou que a dimensão espiritual, quando considerada no âmbito dos cuidados oncológicos, fortalece os laços afetivos entre o trinômio paciente-profissional-acompanhante, além de gerar sentido ao trabalho para os profissionais de saúde^(13)^.

Assim, para transformar a prática profissional, é premente que a temática espiritualidade/religiosidade seja incluída nos currículos dos cursos de graduação e de pós-graduação, a fim de que os estudantes reconheçam a indispensabilidade do assunto em seu campo de atuação, ampliando o entendimento da espiritualidade e da religiosidade, e seu papel tanto para os pacientes quanto para os profissionais de saúde, buscando o cuidado integral e humanizado^(11)^ e um suporte para a saúde do trabalhador. Desse modo, justifica-se o estudo por ser a espiritualidade e a religiosidade consideradas como estratégia de enfrentamento da vida.

## OBJETIVOS

Analisar o uso da espiritualidade/religiosidade por enfermeiros residentes em oncologia na assistência ao paciente oncológico.

## MÉTODOS

### Aspectos éticos

Este estudo respeitou os preceitos éticos no que diz respeito a pesquisas envolvendo seres humanos, conforme preconiza a Resolução nº 466/12 do Conselho Nacional de Saúde. O estudo foi aprovado pelo Comitê de Ética em Pesquisa da instituição proponente e pela instituição coparticipante. Foi disponibilizado aos participantes o Termo de Consentimento Livre e Esclarecido (TCLE) por escrito em duas vias.

### Desenho e cenário

Trata-se de estudo censitário, descritivo, do tipo seccional. A pesquisa teve como local de estudo três hospitais públicos localizados no estado do Rio de Janeiro. Todas as instituições possuíam tratamentos voltados para pessoas vivendo com câncer, bem como programa de residência uni ou multiprofissional em oncologia.

### População ou amostra; critérios de inclusão e exclusão

Participaram da pesquisa 46 residentes, equivalente a 100% da população, sem distinção do ano de curso (primeiro ou segundo), matriculados no programa de residência em oncologia. Critérios de inclusão incluíram enfermeiros residentes matriculados no programa de residência em oncologia do estado do Rio de Janeiro no primeiro ou segundo ano do programa. Critério de exclusão incluíram residentes com menos de seis meses no referido programa devido ser o período de aulas teóricas, sem contato direto com os pacientes, ou que estivessem afastados por licença para tratamento de saúde.

### Medidas de desfecho

Para identificar o perfil sociodemográfico dos enfermeiros residentes, foi utilizado um questionário autoaplicável, elaborado pelos autores, com base em estudos que utilizaram o Protocolo de Avaliação de Riscos Psicossociais no Trabalho. As questões que constituíram as variáveis do estudo foram sexo, idade, estado civil, local de procedência, crença religiosa e se o participante fazia uso da espiritualidade/religiosidade na prática assistencial e, em caso afirmativo, em que situação de trabalho.

### Coleta de dados

A coleta de dados ocorreu no período entre agosto do ano de 2020 e janeiro de 2021, respeitando-se todas as normas de segurança com relação à Covid-19 orientadas pela OMS e Organização Pan-Americana da Saúde (OPAS)^(14)^. A coleta dos dados se deu de forma presencial, utilizando um questionário sociodemográfico, incluindo uma pergunta acerca do uso da espiritualidade e da religiosidade para lidar com suas questões do trabalho. Os questionários foram preenchidos pelos participantes do estudo após a leitura, aceite e assinatura do TCLE.

### Análise estatística

Para analisar os dados coletados, utilizou-se o *software Statistical Package for the Social Sciences* (SPSS), versão 22.0. Foram utilizadas frequências relativas e absolutas para analisar o perfil sociodemográfico dos participantes e para analisar os dados referentes às práticas espirituais e religiosas. Os motivos para as práticas religiosas/espirituais foram distribuídos de acordo com a crença religiosa.

## RESULTADOS

Quanto ao perfil dos residentes (Tabela 1), o sexo majoritário foi o feminino (91,3%), com predominância de idade entre 23 e 29 anos (78,3%), e a maioria declarou estado civil solteiro (80,4%). Quanto à procedência, 73,9% dos participantes eram provenientes da região Sudeste. Em relação à crença religiosa, destacaram-se o protestantismo (34,8%) e o catolicismo (32,6%), e quando questionados sobre o uso da espiritualidade/religiosidade em situações do trabalho, a maioria afirmou fazer uso no seu ambiente laboral (76,1%).

**Tabela 1:** Características sociodemográficas dos enfermeiros residentes de programas de residência em oncologia do estado do Rio de Janeiro, Rio de Janeiro, Rio de Janeiro, Brasil, 2020 (N=46)

Variáveis	n	%
Idade (anos)		
23-29 anos	36	78,26%
30-36 anos	8	17,39%
37-44 anos	2	4,35%
Sexo		
Feminino	42	91,30%
Masculino	4	8,70%
Procedência (região do país)		
Sudeste	34	73,92%
Nordeste	10	21,73%
Centro-Oeste	2	4,35%
Estado civil		
Casado(a)	6	13,04%
Divorciado(a)	1	2,17%
Solteiro	37	80,43%
União estável	2	4,35%
Crença religiosa		
Catolicismo	15	32,6%
Protestantismo	16	34,8%
Espiritismo	4	8,7%
Religiões de origem africana	6	13,0%
Sem religião	5	10,9%
Usa espiritualidade/religiosidade para lidar com suas questões do trabalho?		
Sim	35	76,10%
Não	11	23,90%

Em relação às situações em que os residentes utilizavam a espiritualidade/religiosidade em situações de trabalho, houve referência às situações relacionadas ao cliente (morte, medo, luto e sofrimento, finitude e enfrentamento da doença) e àquelas relacionadas a si próprio (em todas as situações do dia a dia, vulnerabilidade emocional, medo da morte e adoecimento e diferentes sentimentos), conforme apresentado na Tabela 2.

**Tabela 2:** Distribuição das respostas das situações em que os residentes utilizavam a religiosidade e a espiritualidade para prestar assistência ao paciente oncológico (n=35)^^*^^, Rio de Janeiro, Rio de Janeiro, Brasil, 2022

Momentos da prática assistencial do enfermeiro residente	Prática religiosa				
	Católica	Protestante	Espírita/afro	Sem religião	Outras
Situações relacionadas ao cliente					
Morte do cliente	4	2	1	4	3
Medo referido pelo cliente	1	1		1	
Luto e sofrimento				1	
Finitude		1			1
Enfrentamento da doença	1				
Situações relacionadas a si próprio					
Todas as situações (dificuldades do dia a dia/situações difíceis)	2	5	1		
Vulnerabilidade emocional, apreensão e estresse	1	3		1	1
Pedido de ajuda	1	1			
Acontecimento fora do esperado (morte do paciente)	1				1
Medo da própria morte (adoecimento próprio)	1	1			
Forças para continuar		1			
Sentimento de alegria		1			
Sentimento de tristeza		1			
Sentimento de raiva		1			
Sentimento de mágoa	1				

* *Os participantes poderiam ter múltiplas respostas.*

## DISCUSSÃO

A espiritualidade e a religiosidade são interfaces que andam paralelas, e as pessoas que as utilizam possuem melhor bem-estar geral, com menores índices de ansiedade, depressão e comportamentos de risco, como suicídio e uso abusivo de substâncias psicoativas^(15-16)^. No mesmo estudo^(15)^, a espiritualidade abrange a conexão, que pode ser religiosa ou não, com uma realidade não física, conforme percebida pelo indivíduo^(16)^.

Estudo realizado na Holanda indicou a espiritualidade e a religião como elementos essenciais para os cuidados em saúde, em concordância com o presente estudo, apontando que tanto a religião quanto a espiritualidade podem influenciar na vida dos pacientes, bem como na vida dos profissionais de saúde. No local do estudo internacional, as dimensões espiritual e religiosa são vitais para atuação na saúde^(17)^.

Observa-se que a dimensão espiritual e religiosa traz emoções construtivas e, quando vivenciada de maneira positiva, auxilia os pacientes a enfrentar a doença. Dessa forma, contribui para a preservação do estado físico e mental e além do autocuidado, reduzindo o estresse e a ansiedade gerados pelo tratamento em relação aos pacientes oncológicos^(18)^.

No dia a dia, com os residentes, permite-se observar que, além da complexidade do cuidado ao paciente oncológico, ainda possuem o desafio de lidar com suas próprias cargas emocionais, espirituais e religiosas, vivenciando também reflexões acerca de sua formação e atuação profissional na prática do cuidado^(19)^.

Estudo^(20)^ evidenciou a importância da aplicabilidade da espiritualidade e religiosidade na prática clínica, revelando que as mesmas proporcionam força e confiança a pacientes com neoplasia para encarar situações difíceis ao longo do processo da busca pela cura, apontando a essencialidade do uso da fé nessa fase. Esses resultados reforçam ser cada vez mais necessário conhecer as demandas de cuidados espirituais dessas pessoas. Tais dados corroboram o estudo em tela que identificou uma predominância do uso da espiritualidade/religiosidade na atuação no cuidado à saúde pelos enfermeiros residentes. Ademais, grande parte dos indivíduos usa a espiritualidade para lidar com a vulnerabilidade emocional, estresses psicológicos e alterações mentais associadas à ansiedade e a outros sentimentos negativos^(16)^.

A espiritualidade/religiosidade é uma dimensão que transcende os planos carnais e que guia as atitudes dos indivíduos. Estudo transversal, realizado em hospitais do Rio de Janeiro que possuíam programas de residência em oncologia^(3)^ com 53 profissionais atuantes com pacientes críticos, revelou que a maioria apresentava espiritualidade elevada e que tal dimensão exercia forte influência no entendimento e no manejo do processo saúde-doença. Além disso, pôde-se constatar que a espiritualidade/religiosidade era capaz de promover um estado de calma e tranquilidade, assim como melhorias nas dimensões social, emocional e comportamental de quem a exercia.

Investigação americana indicou que experiências espirituais positivas podem melhorar a percepção de uma pessoa sobre a sua autoavaliação de saúde, podendo ser usadas como ferramentas funcionais e positivas pelos profissionais de saúde para a promoção do bem-estar^(21)^. Outra evidência mostra que a espiritualidade está fortemente relacionada ao manejo com momentos estressores, enquanto a religiosidade volta-se para melhores indicadores de saúde como melhor qualidade de vida, redução das taxas de depressão, suicídio e uso de drogas^(22)^. A espiritualidade e a religiosidade são fatores essenciais nos cuidados em saúde, pois contribuem positivamente desde o diagnóstico de uma doença que perpassa por momentos delicados e seu enfrentamento tanto pelo indivíduo quanto pela sua rede de apoio, contribuindo para adesão à terapêutica proposta^(23)^.

Estudo transversal, descritivo e quantitativo, com 31 enfermeiros que trabalharam por pelo menos seis meses em um hospital escola no estado de São Paulo, realizado por meio de amostra por conveniência, mostrou que os participantes relataram que a espiritualidade e a religiosidade interferem em diversos aspectos do ser humano, como a saúde. Além disso, 87% dos participantes relataram sentir a presença de Deus ou do Espírito Santo. Aliado a esse dado, 97% dos enfermeiros declararam acreditar em Deus. Nesse mesmo estudo, 45% dos enfermeiros que participaram da pesquisa descreveram a espiritualidade como a procura de sentido para a sua existência, ressaltando sua essencialidade^(24)^.

Pesquisa descritiva, quanti-qualitativa revelou que 57,6% (n=85) dos profissionais de saúde se consideravam muito ou completamente espiritualizados. Além disso, acreditavam que a religião poderia exercer efeitos positivos na saúde dos enfermos. Ademais, a percepção dos colaboradores sobre a influência da espiritualidade e religiosidade volta-se para a ideia de ser “fonte de conforto” e fortalecimento para lidar com os pacientes com prognósticos reservados^(13)^, ou seja, com probabilidade pequena de melhora.

Pesquisadores ingleses e italianos apontaram que os enfermeiros prestavam regularmente cuidados espirituais aos pacientes, e que esse papel depende da consciência e envolvimento do profissional com a temática. Aqueles que receberam treinamento puderam fornecer um cuidado que ampliasse a confiança do paciente no profissional. No que se refere aos pacientes oncológicos, considerou-se um impacto positivo como resultado^(25)^.

A pesquisa revelou que estratégias de cuidado associadas à espiritualidade diante do processo de morte do paciente oncológico possuíam significado multidimensional e relacional, pois abrangem sentidos, objetivos de vida, significados, crenças e autorreflexão, os quais podem ser observados através da escuta ativa, palavras de conforto e carinho diante da finitude da vida^(13)^.

Estudo exploratório de corte transversal com 55 residentes em um módulo transversal de espiritualidade e integralidade evidenciou que 65,45% dos participantes julgavam necessário a preparação do residente em espiritualidade e que 38,18% realizavam leituras de livros sagrados, preces e orações diariamente^(26)^. Através do ensino, é possível adotar conhecimentos científicos acerca da espiritualidade e religiosidade no contexto de saúde, aprendendo e praticando no cuidado prestado ao paciente, assim como pôde-se encontrar em estudo brasileiro, que analisou o uso da espiritualidade no processo de formação dos residentes, identificando a relevância do uso conceitual associado à prática para agregar valores positivos ao cuidado de enfermagem prestado ao paciente^(26)^.

### Limitações do estudo

As limitações do estudo estão associadas ao fato de ter sido realizado apenas sob a perspectiva de discentes de pós-graduação e em uma única especialidade, a oncologia. Sugere-se que outras pesquisas sejam desenvolvidas para conhecer a percepção dos discentes acerca da pertinência da inserção do tema nos currículos de graduação e de pós-graduação como possibilidades de instrumentalizar o profissional e qualificar a assistência nesta perspectiva junto ao cliente e diante de suas necessidades.

O número de participantes e o tipo do estudo ser censitário podem ser considerados uma limitação; portanto, mais estudos são necessários para corroborar ou refutar os achados apresentados. Sugere-se, ainda, realizar pesquisa multicêntrica, a fim de ampliar a reflexão sobre o uso da espiritualidade/religiosidade na prática dos residentes.

Esta pesquisa apresentou como limitação, ainda, o reduzido número de publicações acerca da temática entre enfermeiros residentes, o que dificultou a comparação com resultados em outras realidades. Isso revela a necessidade premente de investimentos em produção científica nesse campo, a fim de qualificar a espiritualidade e a religiosidade como ferramentas para o cuidado de enfermagem.

### Contribuições para as áreas da enfermagem, saúde ou políticas públicas

Ressalta-se a necessidade da inclusão dessa temática nas matrizes curriculares dos cursos de ensino superior das diversas profissões na área da saúde, para que esses profissionais possam adotar, com mais precisão e embasamento científico, a espiritualidade e a religiosidade em seus planos de cuidados terapêuticos. Além disso, a adoção da espiritualidade e religiosidade no ambiente de trabalho pode contribuir para melhor enfrentamento em situações difíceis.

## CONSIDERAÇÕES FINAIS

A utilização da espiritualidade e religiosidade em ambiente hospitalar traz uma série de benefícios para o trinômio paciente-profissional-acompanhante, desde a melhor aceitação e enfrentamento da doença até a realidade da finitude, bem como a preservação da saúde mental e física dos profissionais de saúde que vivenciam esse cenário com frequência. A espiritualidade/religiosidade são mecanismos utilizados pelos enfermeiros residentes para lidar com as situações vivenciadas pelos pacientes, demandas complexas do trabalho, assim como para autorreflexão das práticas de cuidado.

A situação de maior aplicabilidade da espiritualidade e religiosidade referida pelos residentes é a morte do paciente, independentemente da crença religiosa, uma vez que a finitude e o luto podem ser encarados como falhas da assistência de saúde, pois a formação acadêmica em saúde ainda se espelha no protagonismo do modelo biomédico. Além disso, o benefício volta-se principalmente a situações difíceis e desafiadoras para o residente, momento em que se apega à religiosidade e espiritualidade para amenizar seu sofrimento psíquico e emocional.

Sendo assim, a implementação da temática espiritualidade/religiosidade desde a graduação, bem como sua continuidade nos programas de pós-graduação, torna-se indispensável diante dos resultados positivos que sua prática apresenta.
